# From prostate to the pons: The first reported case of multi‐organ septic emboli and brain empyema from an untreated prostate abscess

**DOI:** 10.1002/ccr3.5018

**Published:** 2021-11-09

**Authors:** Tyler Degener, Samantha Quon, Paul Holtom, Eric Hsieh

**Affiliations:** ^1^ Keck Medical Center University of Southern California Los Angeles CA USA

**Keywords:** prostate abscess, septic emboli

## Abstract

Prostate abscesses in developed countries are becoming increasingly less common. Left untreated these abscesses can lead to numerous complications, including some which are very rare.

## Graphical abstract

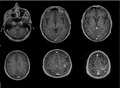

## 1 CASE REPORT

We describe a unique presentation of a patient with an untreated prostate abscess seeding to the brain causing an empyema with severe neurological sequelae and multi‐organ septic emboli.

Prostate abscesses in developed countries are becoming increasingly less common due to the chronicity required for them to develop, as well as the increased availability and access to urgent health care with early antimicrobial treatment in patients with urinary complaints.^1^ Due to these reasons, prostate infections with severe complications are even more rare. Making the definitive diagnosis of a prostate abscess can be challenging for a physician, as early signs and symptoms often mimic other less complicated lower urinary tract infections.^2^ Even more challenging is the treatment of these abscesses due to the lack of cohesive evidence‐based guidelines. At this point, only anecdotal case reports and proposed treatment guidelines have been published in the literature.

Left untreated, a prostate abscess can lead to numerous complications including locoregional spread affecting the function of nearby structures such as the bladder, urethra, penis, and rectum, chronic genitourinary dysfunction requiring subsequent invasive procedures, emphysematous prostatitis, or even sepsis with multi‐organ system failure and death.^3^ At this point in time, there has only been one published case of a prostate abscess metastasizing to the brain causing a complication of a secondary subdural empyema, and in this case, the organism identified was *Staphylococcus aureus*.^4^

Here, we describe a unique presentation of a patient with an untreated prostate abscess seeding to the brain causing an empyema with severe neurological sequelae and multi‐organ septic emboli.

A 47‐year‐old man with a history of uncontrolled diabetes mellitus presented to an outside hospital emergency room with a 3‐week history of general malaise, dysuria, increased urinary frequency and urgency, and subjective fevers. The patient stated that this began when he was camping with his family at a local forest 3 weeks prior to presentation where he admitted to swimming in a freshwater lake. He denied exposure to unsanitary or contaminated food or beverages. He denied hematuria or purulent penile discharge. At the emergency room, he was prescribed a course of amoxicillin, ciprofloxacin, and tamsulosin for a suspected lower urinary tract infection with urethral obstruction as seen with a bladder scan revealing approximately 1 liter of retained urine. After discharge, he continued to have symptoms for another 2 weeks despite finishing the above treatment, and thus represented.

On review of systems, he endorsed recent rigors and mild tingling sensation in his right hand. His physical examination was significant for a temperature of 39.5 degrees Celsius, heart rate of 110 beats/minute, suprapubic tenderness, bilateral flank tenderness, and decreased tactile sensation of his right hand 4 out of 5, and decreased hand‐grip strength in his right hand 4 out of 5. Laboratories on admission were significant for a white blood cell (WBC) count of 16 × 10^3^ cells/mm^3^, hemoglobin of 11.8 g/dl, sodium of 128 mmol/L, chloride of 93 mmol/L, bicarbonate of 23 mmol/L, calcium of 8.2 mg/dl, albumin of 2.3 g/dl, and serum glucose of 377 mg/dl. The calculated anion gap was 12 mmol/L. The urinalysis was significant for protein 30 mg/dl, glucose greater than 1,000 mg/dl, ketones 10 mg/dl, positive leukocyte esterase, negative nitrite, positive for bacteria, and negative for squamous cells.

The patient’s home medications included metformin 500 mg twice a day, insulin glargine 35 units every night, and insulin lispro 12 units with every meal. The patient stated he had not taken any of his medications since the day prior to presentation.

**FIGURE 1:**
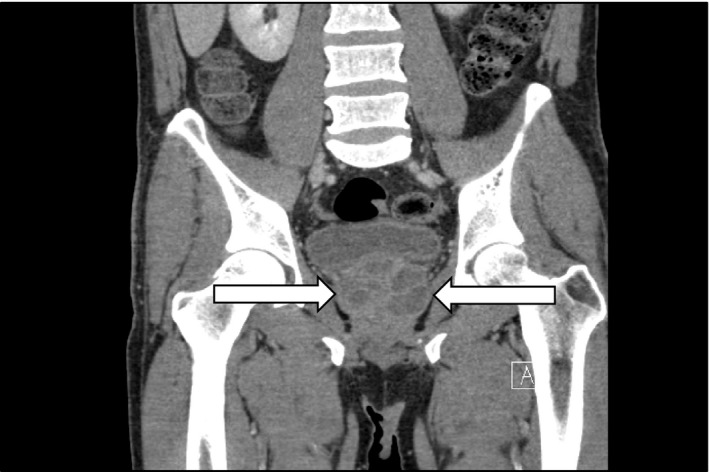
CT Abdomen Pelvis with a large multi‐septated prostate abscess

A computed tomography (CT) scan of the abdomen and pelvis (CTAP) revealed a multi‐septated prostate abscess involving both lobes and the central gland, with the largest discrete component of the left lobe being 2.6 cm and the right lobe being 3.0 cm (Figure 1). The CT also found a thickened bladder wall with ascending urinary tract infection involving both kidneys but without hydronephrosis. Blood and urine cultures were obtained. He was given IV fluid for resuscitation and started on empiric antibiotic therapy with intravenous (IV) ceftriaxone.

Urology was subsequently consulted and recommended a transrectal ultrasound (TRUS) of the prostate with direct visualization of the multi‐septated abscess. The abscess was aspirated and the culture grew *Klebsiella pneumoniae*. Blood cultures from admission did not grow any organism. This *K pneumoniae* isolate had a negative “string test” performed. The patient was switched from ceftriaxone to levofloxacin. On further discussion with the patient, he admitted to unprotected penetrative anal sex with his wife several times over the previous 6 months, which he had denied earlier on admission.

**FIGURE 2:**
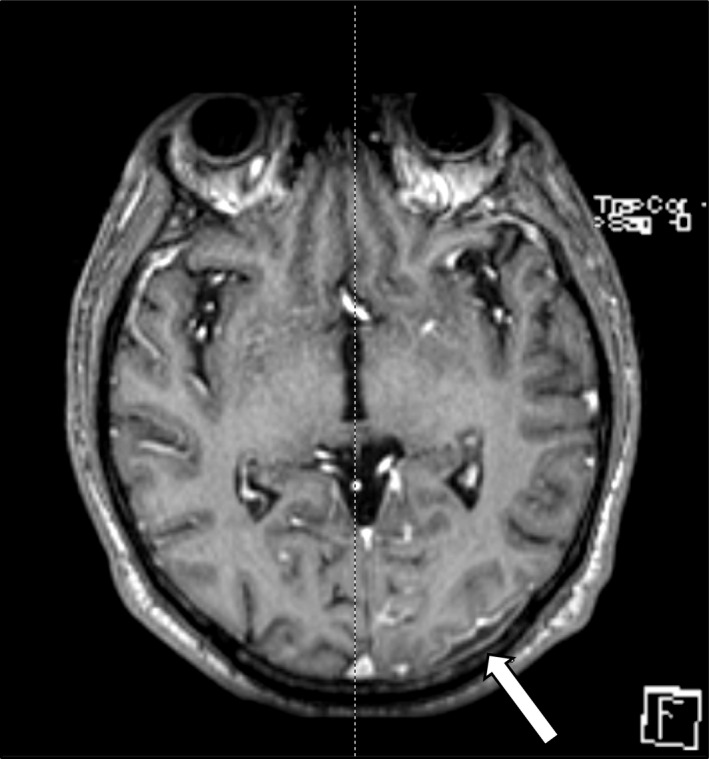
MRI brain with 11mm left occipital fluid collection consistent with a subdural empyema

**FIGURE 3:**
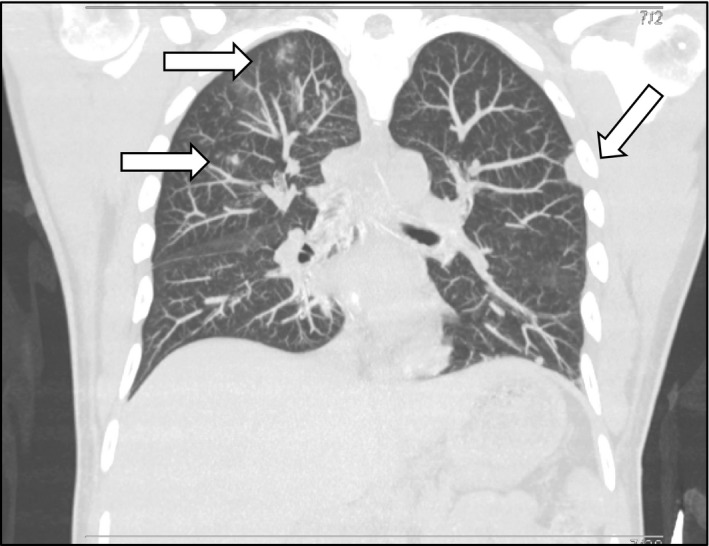
CT Thorax revealing several pulmonary septic emboli (arrows)

The next day, the patient developed acute‐onset right arm weakness, facial droop, and dysarthria. This quickly evolved into a right arm partial seizure with convulsions and lip smacking, which resolved with administration of lorazepam. He was started on levetiracetam for seizure prophylaxis. Brain imaging including CT and magnetic resonance (MRI) revealed numerous scattered acute infarcts in the brain parenchyma, of either septic or embolic origin, as well as a small 1‐2 mm left occipital fluid collection labeled a subdural hematoma (Figure 2). A CT chest was performed showing numerous bilateral cavitary nodules consistent with septic emboli (Figure 3).

Given the clinical and radiographic findings, there was a concern for infective endocarditis in this patient. The infectious diseases (ID) team was then consulted and due to continuing intermittent neurological symptoms recommended starting IV ceftriaxone for double gram‐negative coverage. A transthoracic echocardiogram (TTE) was performed which was negative for endocarditis. A transesophageal echocardiogram (TEE) was performed which was negative for endocarditis however revealed a possible 1‐2 mm atrial septal defect. A repeat CTAP was performed to evaluate for a new focus of infection causing his emboli, which only revealed a decrease in size of the previous largest section of the prostate abscess from 3.0 cm now to 2.4 cm. Urology was re‐consulted for further treatment of the prostate abscess and recommend a transurethral resection of the prostate (TURP) for definitive source control.

**FIGURE 4:**
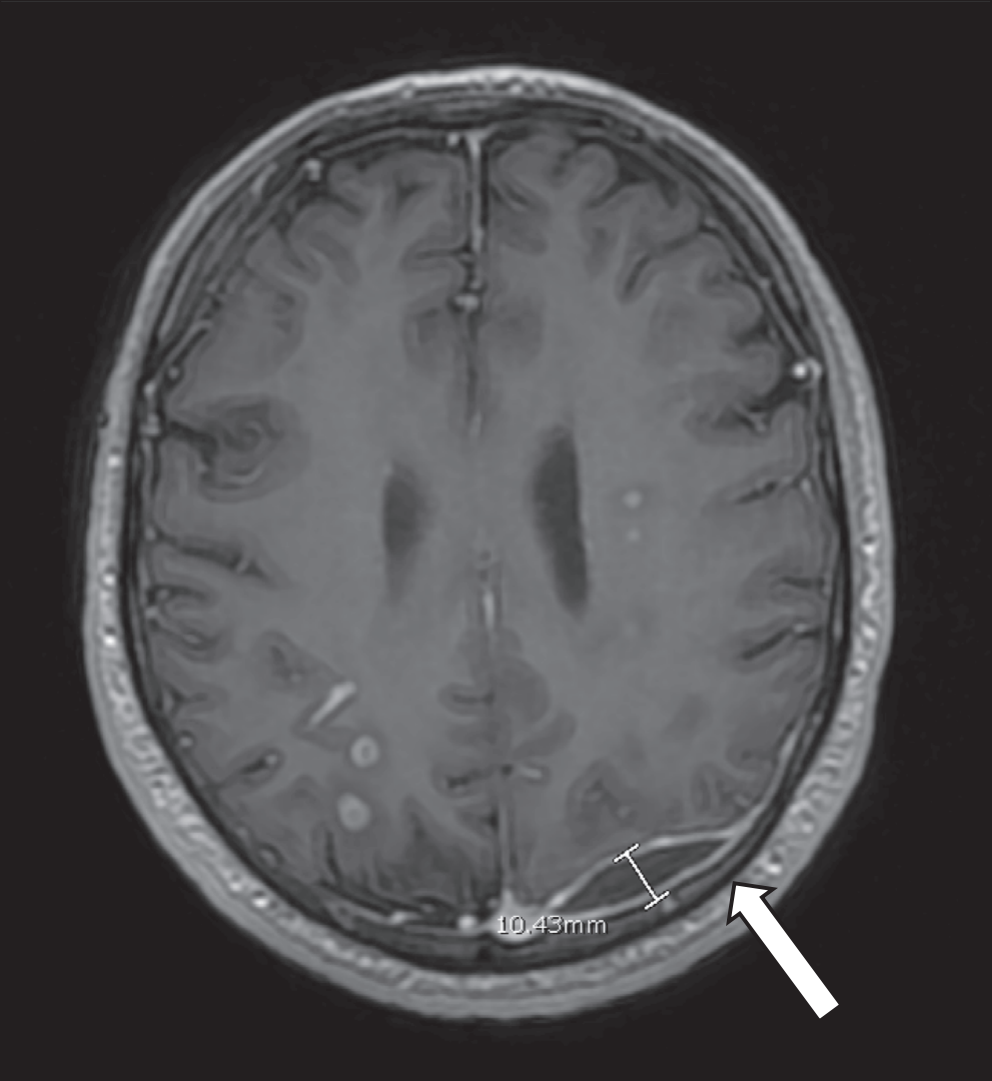
MRI Brain with increase in size of left occipital fluid collection to 11mm, prior to left occipital craniotomy

**FIGURE 5:**
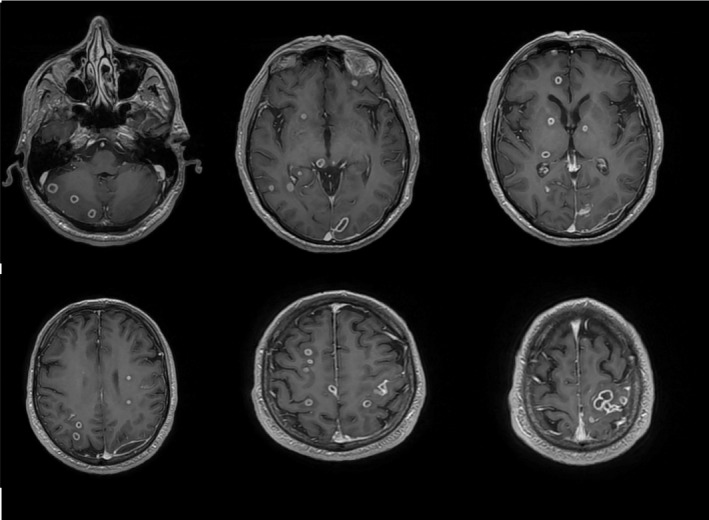
MRI Brain series revealing innumerable septic emboli to brain parenchyma

Directly prior to the planned TURP, the patient had worsening of his neurological symptoms of seizures, lip smacking, and weakness of his right side of his body. He then developed new‐onset vision changes consistent with right inferior homonymous quadrantanopia. Repeat MRI brain revealed an increase in size of the left occipital hematoma to 11 mm and more numerous septic brain emboli. (Figures 4 and 5) Neurosurgery was consulted and reviewed all of the patient’s imaging and re‐diagnosed the patient with having a left occipital brain empyema, likely as a metastasis from the initial prostate abscess, causing mass effect and subsequent neurological symptoms.

There was a large interdisciplinary discussion regarding this patient’s treatment plan. It was agreed that the best course of action would be to obtain source control of his prostate abscess first. Therefore, he underwent an urgent TURP with urology with no complications. Pathology showed tissue necrosis with visualized bacteria and no malignancy. The patient continued on IV ceftriaxone during this time.

Now that there was presumed source control, the next step in the patient’s treatment plan was to tackle his deteriorating neurological status. Once the patient had adequate time to acutely recover from the TURP, he underwent a successful left occipital craniotomy with evacuation of the subdural empyema. The culture from this fluid never grew an organism. After this procedure, all neurological symptoms resolved. Repeat imaging was without evidence of fluid collection in the brain. ID also recommended a cardiac CT to determine if there was a cardiac abscess causing his emboli, which was negative.

The patient was discharged on oral levofloxacin and IV ceftriaxone for a total of 4 weeks of therapy from the date of surgery. There were planned follow‐up appointments with ID, neurosurgery, and urology. He underwent an outpatient tagged WBC/Indium scan for evaluation of another source of infection, which was negative as well.

## 2 DISCUSSION

The current theory as to how a prostate abscess forms is disputed. The common agreement is that all prostate abscesses are directly preceded by an untreated acute prostatitis episode. Due to a disturbance in the genitourinary (GU) tract, there is reflux of urine with eventual stasis and turbulence leading to overgrowth of bacteria causing a pathological infection. This infection then leads to local foci of inflammation, which become walled‐off by the body’s immune response, and form into microabscesses. These microabscesses coalesce into larger clinically significant prostate abscesses.^1^, ^5^ It is estimated that approximately 6% of acute bacterial prostatitis cases will evolve into abscesses.^6^ There are several risk factors a patient may have that predispose him to forming these abscesses. These include advancing age, recent urethral instrumentation or surgery, bladder outlet obstruction, neurogenic bladder disorders, chronic renal failure, liver cirrhosis, diabetes mellitus, and acquired immunodeficiency syndrome.^1^, ^5^, ^7^ In our patient, his only risk factor present was diabetes.

Prior to antimicrobial therapy, the most common cause of prostatitis was *Neisseria gonorrhea*. Now the most common etiology includes *Enterobacteriaceae,* especially *Escherichia coli* (up to 70% in some cases) and *K pneumoniae* (26% in some reports) such as in our presented case.^1^, ^6^ Other less common causes include *Enterococcus faecalis*, *Pseudomonas aeruginosa*, and methicillin‐resistant *Staphylococcus aureus* (MRSA).^1^, ^5^, ^6^ It is important to keep these other less common causes in mind when forming a differential and choosing appropriate antibiotics.

There have been several proposed diagnostic modalities for prostate abscesses including computer tomography (CT) and digital rectal examination (DRE); however, the gold standard has become the transrectal ultrasound (TRUS). The TRUS is able to see abscesses as hypoechoic collections, with a sensitivity ranging between 80 and 100%.^8^, ^9^ CT is also helpful in determining if there have been local or distant advancement of the abscess. MRI has not been recommended thus far.

In regard to treating the prostate abscess, there are no defined guidelines. Based on literature review, the consensus seems to be that a combination of surgical and antimicrobial regimen works best. With regard to antibiotics, empiric treatment with coverage of suspected organisms and eventual tailoring with culture results is recommended.^1^, ^5^ One article by Oshinomi et al studied indications and outcomes of different surgical techniques used to treat prostate abscesses. They found that there are three different approaches: transrectal, transperineal (cutaneous), and transurethral. There were advantages and disadvantages to performing one over the other. Specifically, in our patient’s case, he initially underwent a TRUS with aspiration of the abscess. A disadvantage of this technique includes incomplete drainage, which occurred with our patient. Eventually, the patient had to undergo a transurethral resection of the prostate (TURP). Advantage of this being adequate drainage but with high risk to damaging nearby structures leading to sexual dysfunction, incontinence, and urethral strictures.^10^ It is unfortunate that our patient had to have a TURP performed risking these complications at such a young age, however, he is very much needed source control for his otherwise life‐threatening infection.

There are several noted complications that can occur if a prostate abscess is not treated in a timely manner. These include fistulization into nearby structures such as the urinary bladder, urethra, rectum, or perineum, hematogenous spread to other structures, septic shock, and death.^11^ It has even been documented that untreated prostate abscesses can lead to septic emboli in various places within the body.^12^ Although rare, it is possible for these septic emboli to reach the brain and cause neurological dysfunction such as in our case.

The above‐presented patient developed a very unfortunate and rare sequela of a prostate abscess: a subdural empyema. This is defined as a collection of purulent material between the dura mater and the arachnoid mater. Similar to prostate abscesses, there are no defined treatment guidelines for subdural empyemas. In anecdotal and case reports, however, it seems that the basis of adequate treatment includes both source control via a surgical procedure and antimicrobials. A recently published retrospective study by Widdrington et al looked at 113 cases of subdural empyemas between 2010 and 2016 in an attempt to determine commonalities in management as well as identify factors that could predict negative outcomes. They found that 84% of patients required tailored antibiotic therapy for 6 weeks or more, 91% of patients needed surgical drainage, and 34% needing 2 or more complex repeat surgeries. 97 of the patients (86%) had a microbiologic diagnosis made on culture or pathology, with the most common organisms being gram‐positive bacteria (37% were *Streptococcus anginosus* group and 18% Coagulase‐negative staphylococci), followed by anaerobes and gram negatives (44% and 22%, respectively). They also found that a reduced Glasgow‐Coma Score (GCS), focal neurological deficits, and seizures on presentation were associated with poorer overall outcomes.^13^

This case demonstrates the rare and potentially severe complications of an untreated prostate abscess, as well as the unusual findings of multi‐organ septic emboli and metastasis of primary infection to the brain. These sequelae, which caused alarming neurological symptoms, if recognized, should be acted on urgently with surgical intervention for adequate source control with continued tailored antimicrobial therapy.

## CONFLICT OF INTEREST

All authors have no conflict of interest.

## AUTHOR CONTRIBUTIONS

The authors confirm contribution to the paper as follows: Tyler Degener and Samantha Quon: Draft manuscript preparation. Tyler Degener: Primary author of this manuscript. Paul Holtom and Eric Hsieh: Interpretation of clinical data and case. All authors reviewed the manuscript and approved the final version.

## ETHICAL APPROVAL

All ethics standards and consent were obtained for this manuscript.

## CONSENT

Published with written consent from the patient.

## Data Availability

Data sharing not applicable to this article as no datasets were generated or analyzed for this manuscript.
